# Analysis of College Students' Psychological Education Management in Public Emergencies Based on Big Data

**DOI:** 10.1155/2022/2654437

**Published:** 2022-09-19

**Authors:** Xiaoyu Li

**Affiliations:** School of Energy and Power Engineering, North University of China, Taiyuan, Shanxi 0300051, China

## Abstract

In recent years, college students' psychological problems have occurred frequently, and the early warning of college students' psychological crisis has received social attention. Artificial intelligence and big data, as emerging technologies that have attracted much attention in recent years, have broad application and development space in improving the development of intelligent and refined education in colleges and universities. Applying artificial intelligence and big data to the practice of college students' mental health education plays a very positive role in accurately finding and scientifically solving college students' mental health problems. This paper combs the current application and research of artificial intelligence and big data in college students' mental health education and then clarifies the problems existing in the practical application. Finally, on the basis of in-depth analysis of the characteristics of college students' psychological crisis, the paper designs college students' psychological crisis early warning data collection system from six aspects, including the educational administration system and the access control system. And from the aspects of establishing a multilevel linkage feedback early warning system, building a team of big data technical personnel and mental health education personnel, it puts forward countermeasures for college students' psychological crisis, so as to provide theoretical and methodological support for college mental health management.

## 1. Introduction

With the rapid development of society and big data technology, college students are facing more and more academic, employment, emotional, and economic pressures, which has seriously damaged the original balance ecology of mental health [1]. According to an authoritative survey released by an authoritative organization in China, about 10%~25.4% of college students in many places will have some mental diseases for various reasons and mainly freshmen. These psychological problems of a freshman have become increasingly prominent because of the vicious events such as leaving, suspension, dropping out, and suicide caused by psychological problems. Therefore, the early warning of freshmen's psychological problems has become an important problem that cannot be ignored and urgently needs to be solved in Chinese universities [2].

Although the students entering the university tend to be rational and mature in terms of knowledge and mind, they lack a deep understanding and experience of the reality and have a certain degree of vulnerability in terms of psychology, which leads to their mental health problems due to some bad experiences [3–6]. With the increasing pace of life and learning pressure, mental health has obviously become a prominent problem among college students. Therefore, more and more colleges and universities have incorporated mental health education into the college students' education system, hoping to enhance the ability of college students to discover and solve all kinds of psychological problems of these freshmen or seniors through professional and targeted mental health adjustment. However, the diversity of students' mental states and the lack of teachers' resources for mental health education make it difficult for students' personalized mental health education needs to be effectively met, and mental health education is often reduced to a public knowledge course [7, 8]. Under such circumstances, it is important to ameliorate college teachers' ability to screen, locate, and analyze mental health of students in school with the help of technical means so that teachers can focus more practice and energy on helping students solve psychological problems [9, 10]. Artificial intelligence and big data, as emerging technologies, can precisely meet the needs in this regard.

Since Caplan (1954) put forward the concept of psychological crisis, scholars have conducted a lot of research around psychological crisis. The research related to this paper is mainly reflected in the influencing factors of psychological crisis of college students [11, 12], early warning index system and early warning model of psychological crisis, early warning mechanism system, and early warning intervention management [13–15]. Weist et al. [16] believe that the causes of college students' psychological crisis are complex, and individual factors, family influence, and social environment have an important impact on students' psychological health. Perfect et al. [17–19] used the method of superiority relationship classification to obtain the factors causing psychological crisis of college students.

Reavley et al. [20] analyzed the difference in psychological stress between Chinese and American college students and explored the impact of stress on individual suicidal ideation and depression. Swisher determined the early warning indicator system of psychological crisis from three dimensions: external event indicator, individual information indicator, and social relationship indicator. The development of big data technology has provided a new means for the study of college students' psychological crisis, which has attracted the attention of many scholars. The research in related aspects mainly includes the necessity and feasibility of big data technology in college students' psychological research, implementation methods and model construction [21–23], and application system design. Talbott et al. [22, 24] believe that the traditional psychological crisis early warning system in colleges and universities is inefficient and excessively dependent on clinical scales. Therefore, big data thinking should be integrated into the psychological crisis early warning work in colleges and universities. Chen et al. [25] proposed an innovative path for college students' mental health education in the context of big data. Guedes et al. [24] introduced the concept of big data into the field of mental health education in colleges and universities, analyzed the mental health data of college students in detail, and established a model of mental health data feedback system. To sum up, the research on college students' psychological crisis early warning under the background of big data will be a new trend in the future. We should use big data technology and combine various data systems on campus to build a psychological crisis early warning system.

Existing studies have found that some scholars have put forward the conceptual model and mechanism of psychological crisis management based on big data [26, 27], but the operability is not strong. Based on this, on the basis of analyzing the concept and characteristics of college students' psychological crisis, combined with the problems and the needs of college students' psychological health education, this paper puts forward suggestions on deepening the application of technology in college students' psychological health education, systematically builds a college students' psychological crisis early warning system under big data, and puts forward scientific, targeted, and effective countermeasures and reference methods for college students' psychological crisis, in order to provide theoretical and methodological support for college mental health management.

## 2. The Concept and Characteristics of Young Students' Psychological Crisis

### 2.1. Concept

Academic circles have different views on the specific concept of psychological crisis. At present, the definition of the connotation of psychological crisis mainly meets the following conditions: ① psychological crisis is the manifestation of the conflict between ideal and reality in people's daily life, ② psychological crisis is a variety of negative emotions that individuals cannot find a way to deal with or solve sudden things, and ③ psychological crisis is the psychological and physiological imbalance caused by individuals' failure to cope with external interference. To sum up, college students' psychological crisis refers to the psychological imbalance caused by college students' failure to respond in time after encountering emergencies and then showing high tension, anxiety, confusion, and other negative emotions.

### 2.2. Characteristics

University is an important stage of life. College students who are separated from the supervision and assistance of their parents need to face the problems of professional study, making friends, employment, postgraduate entrance examination, etc., and their life in university is collective and free. Therefore, this paper believes that compared with other groups, college students' psychological crisis has the following characteristics:

1. Concealment: the management of the university is relatively free. The teachers, counselors, and class teachers have limited contact with the students in the class, so they cannot know the real psychological status of the students. However, the classmates who contact the most often do not have the ability to identify psychological problems. When psychological problems cannot be solved in a timely manner, psychological problems tend to accumulate into a big one and turn into a psychological crisis
2. Danger: when college students encounter psychological crisis, it is usually difficult to complete psychological repair alone. Without the help of the outside world, they are prone to fall into the vortex of self-denial and even to harm others and commit suicide
3. Sudden crises are often unexpected and uncontrollable. For example, the SARS epidemic in the spring of 20d3 caused great panic to the college students in Beijing at that time. At that time, the normal study and life rank were disrupted; the destruction of the order, the departure of classmates and friends, and the isolation of the campus have all caused serious psychological crisis to college students
4. The arrival of helplessness psychological crisis often makes people feel at a loss, and people's future plans are threatened and destroyed. Because the previous coping style cannot cope with the crisis and the social support system is not perfect, college students often feel helpless and desperate

## 3. Challenges Faced by Big Data Application in Related Fields

### 3.1. Insufficient Attention to Technology Application

These years, the extensive use of artificial intelligence and big data in the business field has gradually deepened the perception and recognition of the practical value of these emerging technologies by colleges and teachers, but few colleges and universities really practice from the perspective of technology in related fields and psychological education for young students. On the one hand, this problem of low attention limits the effective application of scientific research achievements in big data and other place to the practice of young students' mental health guidance, making the mode of young students' mental health guidance too old and ineffective. On the other hand, it also weakens the research and practical exploration of some college teachers on the application of artificial intelligence and in young students' mental health education and lacks corresponding conditional support, which cannot change the good to promote this education.

### 3.2. Difficult Technical Development

Big data is a universal technology. Research shows that these technologies can be applied to college students' mental education. There is a lack of clear and mature content guidance on how to apply, which makes the application of artificial intelligence and big data in young students' mental education more difficult. Meanwhile, the application of big data in young students' health education involves the integration of professional technologies such as big data and professional disciplines such as psychology, which requires that personnel involved in technology development not only understand the technical growth of young students' mental health education system or platform but also understand the professional knowledge of psychology. Obviously, such talents are scarce among the new college talents in this period; this makes the application of big data in college students' mental health education face greater technical difficulties.

### 3.3. Data Sharing Is Difficult

In the application of 5g network, artificial intelligence, cloud computing, big data, “Internet of things+,” and other information technologies, education management methods, models, and systems have been innovated and strengthened, and the status and value of management data in daily education management have been enhanced. Meanwhile, educational institutions and schools have gradually strengthened interschool exchanges and cooperation and paid attention to the sharing and circulation of education management data. However, due to the diversity and large number of big data in education management, it is difficult to achieve effective compatibility and sharing. For example, in the process of software construction and hardware construction, there are obvious differences among different cities, regions, and schools, resulting in many problems in the process of data transmission and sharing.

### 3.4. The Effect of College Students' Participation Is Not Good

Although the application of artificial intelligence and big data can improve the pertinence and accuracy of college students' psychological education, the premise to achieve this effect is to have enough data information so that various models and systems can operate on this basis. In other words, college students' participation in psychological education activities is the premise and foundation for big data to play its role in your field. At present, few students are able to take the initiative and participate in relevant activities in this field. Some students are worried about the leakage of their personal information, and they are not willing to participate in this kind of mental health education. This poor participation results in the poor role of technology in the mental health education of college students, significantly affecting the application of artificial intelligence and big data.

## 4. Construction of Psychological Crisis Early Warning System Based on Big Data Technology

There are a large number of students in universities. Students' big data belongs to scattered information resources, and its information value is extremely limited. However, using big data technology for in-depth mining and comprehensive utilization can transform these scattered information into valuable information and finally achieve the effect of information serving universities and students.

### 4.1. Collection of Psychological Early Warning Data

University is a process in which we gradually change from a student to a professional. During this period, we will inevitably experience various hardships and pressure will be generated in all aspects. Due to the limited psychological endurance and digestion ability, college students are prone to psychological imbalance. After reading the literature and in-depth research, this paper analyzes the following psychological warning data sources. Class attendance and examination results of each semester: take the professional course of a college student with 10 classes per week as an example, through random sampling survey of 8000 students in the school, the number of students in the sampling survey is 100, and the results of 100 students are summarized and averaged. The survey results are shown in Table 1.

**Figure 1:**
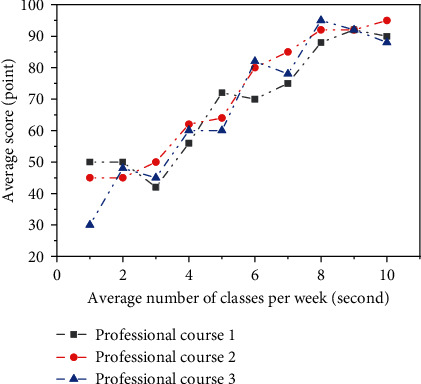
Psychological warning data—relationship between grades and class times.

Figure 1 describes the relationship between college students' attendance and their average scores. From the figure, it can be seen that if students are always late or absent from class, their average scores will be lower, and there is basically a positive correlation. However, for some special cases, some students still have high scores despite less class times; it is necessary to find out the reasons, whether they are part-time or have an improper weariness of learning due to economic difficulties. Usually, the school will have a relatively strict attendance system, requiring the class study committee to arrive before each class, count the list of students who are late and absent, and submit it to the school study department every weekend. Then, the learning department will hand it over to the counselors. The Academic Affairs Office will keep the academic results of each student in each semester, and the data is easy to collect.

**Figure 2:**
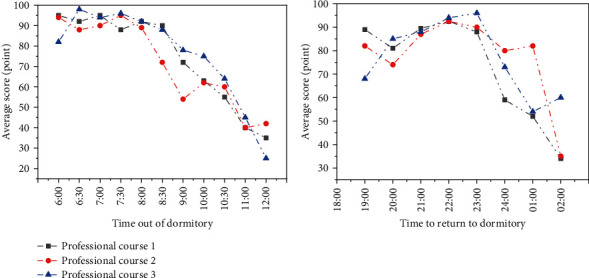
Dormitory access control system data—relationship between score and bedtime.

For dormitory access control system data, the survey statistics are shown in Figure 2. Taking the time of boys' arrival at bedtime as an example, two main data are grasped. One is the earliest time to swipe the card every day, and the other is the time to return to the dormitory at night. If the time of going out of the dormitory every day is after 11:00, such students are very likely to have the bad habit of staying in bed, staying up late, and getting up late. Such students are prone to feel confused about college life, which deserves attention. The late return to the dormitory but before the deadline of access control may be due to the high pressure of learning, part-time work, etc. If you frequently return to school in the early morning, you need to pay special attention.

In addition, the consumption data of school campus card and the payment of educational administration system can also reflect the mental health of students. Understand the fluctuation of students' consumption through the campus card and judge whether the students' life is stable. From the academic affairs office, we can learn about the payment of students' tuition fees. Most of the students who have been in arrears for a long time are in financial difficulties and face the pressure of the school to urge them to pay their tuition fees. However, we cannot rule out that some students use their tuition fees for other purposes, which leads to arrears. In any case, they need to bear the pressure psychologically. Data was published by students on major social networking sites. The Internet is the main way for college students to vent their emotions. The main social software we use are QQ, WeChat, and Weibo. With the consent of students, we regularly obtain valuable information related to mental health through big data technology.

Interpret the psychology of students from the perspective of psychology. There will be different troubles at different stages of the university. Generally speaking, in the freshman stage, it is easy to be far away from home, have no friends, feel lonely or do not like your major and school, and need a process of adaptation to the life on the university campus. In sophomore and junior years, emotional problems, such as interpersonal problems or love problems, are more likely to occur. In the third and fourth years of the university, the students are about to graduate. The biggest pressure of the students who take the postgraduate and civil service examinations comes from their heavy learning tasks. The students who are directly employed often have no confidence in their abilities and worry about their future development. In general, different students should have different problem preferences and should make full use of the file information of the students when they enter the school and keep the data updated in four years. At the same time, all data should be electronic and sent to the mental health education center to facilitate the analysis of the psychological crisis early warning system.

### 4.2. Construction of Psychological Crisis Early Warning System Based on BP Neural Network

#### 4.2.1. Principle of BP Neural Network

**Figure 3:**
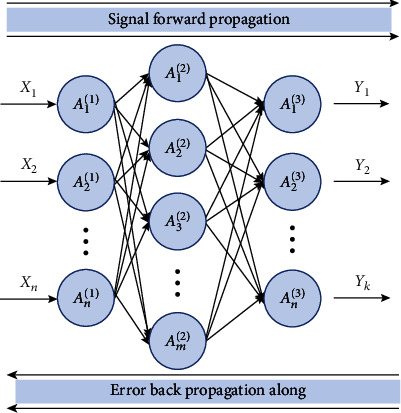
BP neural network structure diagram.

From the principle of the neural network, it can be found that the initial goal of the network is to simulate the working mechanism of human brain neurons. By setting up a series of neurons in the network to sense external signals or some stimulating behaviors, these perceived signals are transmitted to the internal perception unit through a neuron called input gate and propagate along the forward direction. At the same time, in the process of propagation, back feedback the propagation error to adjust the model, so as to establish a network in which the skill is passing through the propagation signal and the error can be transmitted back to form a BP neural network. Its network structure is shown in Figure 3.

In this picture, *n* and *k* represent the number of input signals and output results, respectively; *θ* represents the connection weights between the layers, including the weights between the input layer and the hidden layer, the weights between the hidden layer and the hidden layer, and the weights between the hidden layer and the output layer; and *a*_*j*_^(*i*)^ represents the excitation value, where *i* represents the network layer. Therefore, the BP neural network can be regarded as a nonlinear function. Through repeated learning, it can adjust the connection weights between multiple neurons and discover the complex rules within the data and has good robustness and fault tolerance.

#### 4.2.2. BP Neural Network Operation Process

**Figure 4:**
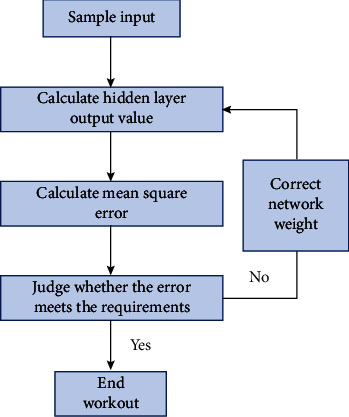
BP neural network operation flowchart.

This section introduces the operation process of the BP neural network in combination with its learning algorithm and takes the three-layer BP neural network as an example. The specific process is shown in Figure 4.

Suppose that the input layer neuron node of the BP neural network is *x*_*n*_, (*i* = 1, 2, ⋯, *N*), the hidden layer neuron node is *z*_*j*_, (*j* = 1, 2, ⋯, *J*), and the output layer neuron node is *y*_*k*_, (*k* = 1, 2, ⋯, *K*). The connection weights between the hidden layer and the input layer and the output layer are *μ*_*nj*_ and *ω*_*jk*_, respectively. The interlayer thresholds are represented by *γ*_*j*_ and *θ*_*k*_, respectively, and the expected output value of the output layer is *d*_*k*_.

**Step 1 .**

Set initialization parameters and learning parameters and input sample data. Initialization parameters include connection weight *ω*_*jk*_ and thresholds *γ*_*j*_ and *θ*_*k*_. Learning parameters involve learning rate *η*, *ε* transfer function, allowable error 6, training times *M*, and the number of neuron nodes at each layer.

**Step 2 .**

Secondly, according to the optimization results of the previous step, the network function is used to calculate the values of each layer. The output value of the layer can be calculated from the formula in *z*_*j*_ below, and the output value of the layer can be calculated from the formula in *y*_*k*_ below.

(1) $$\begin{array}{c} z_{j}=f\left(\overset{N}{\underset{n=1}{\sum }}\mu_{nj}x_{n}-\gamma_{j}\right), \end{array}$$

(2) $$\begin{array}{c} y_{k}=f\left(\overset{J}{\underset{j=1}{\sum }}\omega_{jk}z_{j}-\theta_{k}\right). \end{array}$$

**Step 3 .**

Next, in the third step, after solving the values in the second step, we need to use the following formula to solve the mean square error:

(3) $$\begin{array}{c} E=\frac{1}{2}\overset{K}{\underset{k=1}{\sum }}{\left(d_{k}-y_{k}\right)}^{2}=\frac{1}{2}\overset{K}{\underset{k=1}{\sum }}e_{k}^{2}. \end{array}$$

**Step 4 .**

Judge whether the BP neural network training is over. When the mean square error *E* is less than the allowable error *ε* (network convergence) or when the training times exceed the preset *M* value (the network cannot converge), the training ends. When the mean square error*E*is greater than the allowable error*ε*, it is necessary to calculate the output layer correction error *δ*_*jk*_ and the hidden layer correction error *δ*_*nj*_ according to

(4) $$\begin{array}{c} \delta_{jk}=-\frac{\partial E}{\partial \left(\sum_{j=1}^{J}\omega_{jk}z_{j}-\theta_{k}\right)}=-\frac{\partial E}{\partial y_{K}}\frac{\partial y_{K}}{\partial \left(\sum_{j=1}^{J}\omega_{jk}z_{j}-\theta_{k}\right)}=\overset{K}{\underset{k=1}{\sum }}\left(d_{k}-y_{k}\right)y_{k}\left(1-y_{k}\right), \end{array}$$

(5) $$\begin{array}{c} \delta_{nj}=-\frac{\partial E}{\partial \left(\sum_{n=1}^{N}\mu_{nj}x_{n}-\gamma_{j}\right)}=-\frac{\partial E}{\partial y_{K}}\frac{\partial y_{K}}{\partial z_{j}}\frac{\partial z_{j}}{\partial \left(\sum_{n=1}^{N}\mu_{nj}x_{n}-\gamma_{j}\right)}=\delta_{jk}\omega_{jk}z_{j}\left(1-z_{j}\right). \end{array}$$

**Step 5 .**

Modify the weights and thresholds of neurons at each layer. Modify the connection weights according to formulas (6) and (7) and modify the thresholds of these two layers according to formulas (8) and (9). When the weights and thresholds are updated, continue with Step 2 until the end of the training.

(6) $$\begin{array}{c} \Delta \mu_{nj}=-\eta \frac{\partial E}{\partial \mu_{ij}}=-\eta \frac{\partial E}{\partial \left(\sum_{n=1}^{N}\mu_{nj}x_{n}-\gamma_{j}\right)}\frac{\partial \left(\sum_{n=1}^{N}\mu_{nj}x_{n}-\gamma_{j}\right)}{\partial \mu_{nj}}={\eta \delta }_{nj}x_{n}, \end{array}$$

(7) $$\begin{array}{c} \Delta \omega_{jk}=-\eta \frac{\partial E}{\partial \omega_{jk}}=-\eta \frac{\partial E}{\partial \left(\sum_{j=1}^{J}\omega_{jk}z_{j}-\theta_{k}\right)}\frac{\partial \left(\sum_{j=1}^{J}\omega_{jk}z_{j}-\theta_{k}\right)}{\partial \omega_{jk}}={\eta \delta }_{jk}, \end{array}$$

(8) $$\begin{array}{c} \Delta \gamma_{j}=-\eta \frac{\partial E}{\partial \gamma_{j}}=-\eta \frac{\partial E}{\partial \left(\sum_{n=1}^{N}\mu_{nj}x_{n}-\gamma_{j}\right)}\frac{\partial \left(\sum_{n=1}^{N}\mu_{nj}x_{n}-\gamma_{j}\right)}{\partial \gamma_{j}}=-{\eta \delta }_{nj}, \end{array}$$

(9) $$\begin{array}{c} \Delta \theta_{k}=-\eta \frac{\partial E}{\partial \theta_{k}}=-\eta \frac{\partial E}{\partial \left(\sum_{j=1}^{J}\omega_{jk}z_{j}-\theta_{k}\right)}\frac{\partial \left(\sum_{j=1}^{J}\omega_{jk}z_{j}-\theta_{k}\right)}{\partial \theta_{k}}=-{\eta \delta }_{jk}. \end{array}$$

#### 4.2.3. System Construction Based on BP Neural Network

**Figure 5:**
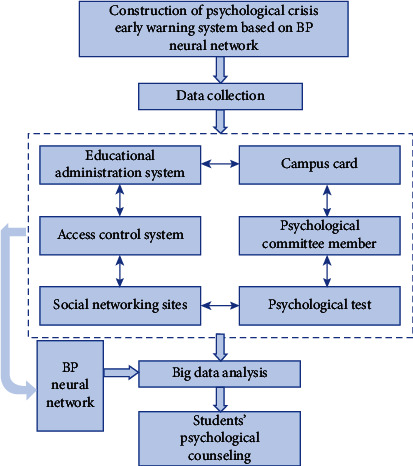
Construction of psychological crisis early warning system based on BP neural network.

Through the preliminary work, the database of the psychological crisis early warning system has six aspects: attendance, academic performance of each semester, time of going in and out of the dormitory, consumption of campus cards and whether the tuition fees are paid on time, data from social networking sites, information reflected by class psychological committee members, and mental health diagnosis scale. Then, it can predict the psychological status of students through big data processing, mining, and analysis. After the prediction results are obtained, they are fed back to the school mental health education center. The physical and mental education center cooperates with the counselor. The counselor contacts the parents and uses professional psychological counseling methods to help the students with psychological crisis. The overall process is shown in Figure 5.

#### 4.2.4. Result Analysis of BP Neural Network Model Training Set

**Figure 6:**
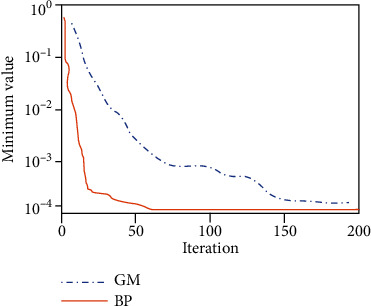
Change of prediction error of two models.

**Figure 7:**
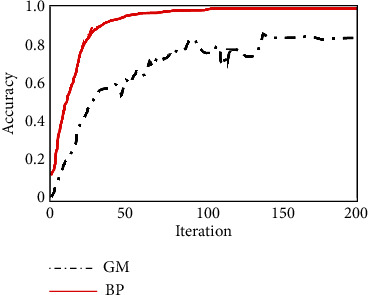
Prediction accuracy of two models.

Through Section 4.2.3, we have completed the construction of the psychological crisis early warning system based on the BP neural network model, and through the analysis of the campus psychological database about the students' academic performance in each semester, the time of entering and leaving the dormitory, the consumption of the campus card (average daily consumption expenditure), and whether the tuition is paid on time, the situation is reflected by the class psychological committee. The data of the six aspects of mental health diagnosis scale (diagnosis times in a year) (respectively, recorded as *a*, *b*, *c*, *d*, *e*, and *f*) (the results of some of the called data are shown in Table 2) and the above results are used in the training of the BP neural network model to predict the students' psychological conditions. At the same time, in order to compare the reliability of the model in this paper, the data in this paper are used in the conventional model (gray prediction model) for comparative analysis. The prediction accuracy and model prediction error of the two models are taken as the evaluation indexes. The prediction results of the two models are shown in Figures 6 and 7, respectively.

It can be seen from the change of the prediction error of the two different models with the number of iterations in Figure 6 that the test error of the two models gradually decreases with the increase of the number of iterations of the model, and both can reach a small value. However, the difference is that the BP neural network prediction model constructed in this paper is better than the gray prediction model of the comparison model in terms of the prediction error of the model and the number of iterations of the model. Among them, the error of the BP neural network quickly tends to be stable after traversing about 60 iterations, and the final error of the model is close to 10-4, which has reached a minimum error. It can be said that the test error of the model has little impact on the results. In contrast, the prediction effect of the gray prediction model is poor. The test error of the model is not only much larger than that of the BP neural network model but also the number of iterations is more than that of the BP model. It takes more than 150 iterations before the model error tends to be stable. Therefore, compared with the gray prediction model, the training speed and error control ability of the students' psychological change prediction model based on the BP neural network model constructed in this paper are better than those of the gray prediction model.

Figure 6 shows the change of the prediction accuracy of the two different models with the number of iterations. It can be seen that the prediction accuracy of the two models gradually increases with the increase of the number of iterations of the model. However, the difference is that the BP neural network prediction model constructed in this paper is better than the gray prediction model of the comparative model in terms of the prediction accuracy of the model and the number of iterations of the model. Among them, the error of the BP neural network can be rapidly improved to above 0.95 after 100 iterations, and the maximum accuracy is as high as 0.976. In contrast, the prediction accuracy of the gray scale prediction model is poor. The prediction accuracy of the model is not only much lower than that of the BP neural network model but also the number of iterations is more than that of the BP model. After more than 150 iterations, the prediction accuracy of the model can reach the maximum value, and the maximum value does not exceed 0.8. The training speed and prediction accuracy of the student's psychological change prediction model based on the BP neural network model constructed in this paper are better than those of the gray prediction model.

In summary, it can be found that the student psychological prediction model based on the BP neural network constructed in this paper is not only better than other models in terms of training speed and training error but also more accurate in predicting students' psychological conditions, with the highest accuracy rate close to 1, which indicates the effectiveness and superiority of the model constructed in this paper.

## 5. Countermeasures for College Students' Psychological Crisis Based on Big Data Technology

At present, the development of the psychological crisis early warning system in Chinese colleges and universities has not formed a complete system, and various problems emerge in endlessly, which have seriously affected the response efficiency of colleges and universities to the psychological crisis of college students. Through research, this paper puts forward the following suggestions and measures according to the current situation.

### 5.1. Establish Multilevel Linkage Feedback Early Warning System

Mental health education centers, schools, colleges, parents, classes, and dormitories should unite to establish a six-level early warning system for psychological crisis and cooperate at all levels to help students overcome psychological problems. The school is responsible for overall planning. The mental health education center is responsible for providing professional psychological assistance. College mainly refers to the teachers led by counselors and class teachers, who are responsible for discovering the psychological changes of students at the first time. The responsibility of the family is not only limited to the students' money input but also needs to bear the responsibility of psychological cultivation. The students in the class, especially the psychological committee members, should help the students with poor psychological conditions and regularly feedback the students' psychological conditions to the counselors and teachers. The six systems shown in the figure not only represent the original information we have obtained but also the most effective tool for counseling and treating young students' psychological weaknesses. In this system, there are many factors that can produce effects, including family, school, and society. Therefore, for the physical and mental problems of young students, the three must work together to make this system effective.

### 5.2. Encourage Students to Actively Participate in the Construction of Psychological Crisis Early Warning

Usually, it is difficult for people to talk about psychological problems, and they show negative or unconcerned feelings about psychological tests and other psychological problems. However, due to the extensive use of mobile phones by college students, mobile phones do not need face-to-face communication, which reduces more psychological barriers. Through the app, college students are encouraged to actively participate in the psychological crisis early warning system and actively participate in the six functions of the app, including index data query, mental health test, mutual assistance in the forum, daily mood record, psychological status scoring, and psychological consultation appointment so that students can open their hearts to psychological problems and no longer repel.

### 5.3. Optimize Technology Application Mode

For a long time, college students have shown obvious sensitivity to their own psychological problems and are unwilling to talk about them too much, which makes it difficult to carry out young students' physical and mental education in an open and public way. Artificial intelligence, big data, etc. can be used with advantages of other platforms, such as the neural network model, so that colleges and universities and teachers can develop personalized young students' physical and mental health education platform with the network as the carrier so that students can log in from this interface at any time and at any place for content understanding and activity participation and truly eliminate the worry of young students about physical and mental education. In the actual operation of these technologies, colleges and universities can develop a physical and mental education platform for all young students and can also develop a personalized mental health education platform according to students' different age, gender, grade, major, and other conditions, meanwhile creating a better learning environment for them and conditions for students to participate in the application of technology. Meanwhile, we need to pay attention to the timely and objective feedback of the results of mental health education so that the young students can really feel the advantages and values of artificial intelligence and big data in mental health education and gradually eliminate the exclusion and resistance of students. For example, teachers can design some personalized questionnaire questions with the help of the system and make corresponding adjustments to the design of young students' mental education platform according to the final feedback results of students, so as to make the platform more appropriate to their lives and changes in their psychological activities.

### 5.4. Cultivate a Team of Psychological Talents with Big Data Technology

In practice, the mental education center in our universities lacks talents who master data technology, which is one of the important reasons why the psychological crisis early warning system in colleges and universities cannot be improved. This team needs compound talents who not only understand physical and mental education knowledge but also master big data technology. By establishing a university mental health education center with both physical and mental education talents and data technology talents, we can maximize the application of data technology and alleviate the bottleneck of traditional psychological early warning methods.

### 5.5. Integrate Data Technology Development Resources

The application of big data in college students' mental health education is an innovative content, which requires the participation of professional technical personnel and psychological experts and needs to be continuously developed and improved. Considering the fact that the research and practice ability of colleges and universities in this area is weak at present, we can enhance the ability of technology development and break through the difficulties of technology application through resource combination and other means. In the aspect of resource integration, it can be carried out in different ways, such as cooperation among universities and cooperation between universities and enterprises. For example, strong universities can carry out special research on the application of artificial intelligence and big data to college students' mental health education through the combination of strong and strong. Through the cooperation of technical experts and psychological experts, they can make up for the shortcomings of universities in technology development and application and create a technical platform that truly meets the needs of college students' mental health education.

In addition, colleges and universities can also carry out technical cooperation with enterprises or organizations outside the university with strong technical strength, integrate their professional advantages in psychology with the advantages of technological development of other organizations, and promote the transformation of relevant technological ideas into technological achievements. At the same time, considering the personalized characteristics of college students' mental health, the relevant subjects should not only pay attention to the development of the early-stage technology platform but also carry out regular technical maintenance and updating according to the follow-up college students' mental health education practice so that the application of artificial intelligence and big data technology can closely follow the development of college students' mental health education practice. For example, colleges and universities can regularly optimize the model in the college students' mental health assessment system to optimize the matching degree between the model and the data in the database.

### 5.6. Ensure the Data Security of Mental Health Education Center

The university stage is a critical time for people's growth. College students at this stage are extremely sensitive and unstable and dare not touch topics like psychological problems and psychological crises easily. In essence, colleges and universities establish a psychological crisis early warning system to help students. After obtaining the psychological status information of students, they should ensure the absolute security of the information and limit the number of APP information input ports. Counselors, class teachers, and psychological committee members should strictly keep students' secrets, so as to avoid the effect contrary to the original purpose.

## 6. Conclusion

In view of the current situation of frequent psychological crisis of college students, this paper uses big data technology to make up for the shortcomings of traditional psychological crisis warning methods. After collecting the six aspects of psychological early warning data, the big data technology is used to analyze, mine, and predict the data. The mental health education center and teachers carry out psychological intervention on the students according to the prediction results. At the same time, a BP neural network model for predicting psychological crisis is constructed, and the model is compared with the commonly used gray prediction model. The results show that the psychological crisis early warning system constructed based on big data technology and BP neural network can quickly and accurately judge the psychological status of students by using the timeliness of big data technology, with the highest prediction accuracy of 0.976. This shows the validity of the model and the necessity of research. In addition, in view of the problems existing in the current education management of college students, this paper puts forward six countermeasures based on the analysis results: (1) establish a multilevel linkage feedback early warning system, (2) encourage students to actively participate in the construction of psychological crisis early warning, (3) optimize the application mode of technology, (4) cultivate a team of psychological talents with big data technology, (5) integrate data technology development resources, and (6) ensure the data security of the mental health education center to ensure the mental health and physical and mental safety of college students.

## Figures and Tables

**Table 1:** Attendance of three professional courses of college students and examination results of each semester.

Attendance times	Professional courses		
	1	2	3
10	90.0	95.7	87.6
9	92.1	91.6	91.5
8	88.4	92.3	95.3
7	75.3	84.7	77.6
6	70.1	80.2	81.7
5	71.7	63.9	60.3
4	55.8	61.8	59.7
3	42.0	50.4	45.0
2	49.8	45.0	48.0
1	50.1	44.6	30.2

**Table 2:** Mental health database of campus students.

Students	*a*	*b*		*c*/RMB	*d*	*e*	*f*
		Enter	Out				
1	84.16	22:17	7:26	21.0	Yes	Healthy	1
2	75.27	23:48	8:25	20.5	Yes	Healthy	1
3	82.14	23:02	8:44	16.0	Yes	Healthy	1
4	52.29	24:51	11:21	30.0	Yes	Unhealthy	6
⋮	⋮	⋮	⋮	⋮	⋮	⋮	⋮
*N* − 1	63.98	20:16	10:25	24.0	Yes	Commonly	1
*N* − 2	77.21	22:01	6:54	26.0	Yes	Healthy	1

## Data Availability

The experimental data used to support the findings of this study are available from the corresponding author upon request.
